# Vision-Based Road Rage Detection Framework in Automotive Safety Applications

**DOI:** 10.3390/s21092942

**Published:** 2021-04-22

**Authors:** Alessandro Leone, Andrea Caroppo, Andrea Manni, Pietro Siciliano

**Affiliations:** National Research Council of Italy, IMM—Institute for Microelectronics and Microsystems, 73100 Lecce, Italy; andrea.caroppo@cnr.it (A.C.); andrea.manni@le.imm.cnr.it (A.M.); pietro.siciliano@le.imm.cnr.it (P.S.)

**Keywords:** road rage detection, ADAS, face detection, facial expression recognition, transfer learning

## Abstract

Drivers’ road rage is among the main causes of road accidents. Each year, it contributes to more deaths and injuries globally. In this context, it is important to implement systems that can supervise drivers by monitoring their level of concentration during the entire driving process. In this paper, a module for Advanced Driver Assistance System is used to minimise the accidents caused by road rage, alerting the driver when a predetermined level of rage is reached, thus increasing the transportation safety. To create a system that is independent of both the orientation of the driver’s face and the lighting conditions of the cabin, the proposed algorithmic pipeline integrates face detection and facial expression classification algorithms capable of handling such non-ideal situations. Moreover, road rage of the driver is estimated through a decision-making strategy based on the temporal consistency of facial expressions classified as “anger” and “disgust”. Several experiments were executed to assess the performance on both a real context and three standard benchmark datasets, two of which containing non-frontal-view facial expression and one which includes facial expression recorded from participants during driving. Results obtained show that the proposed module is competent for road rage estimation through facial expression recognition on the condition of multi-pose and changing in lighting conditions, with the recognition rates that achieve state-of-art results on the selected datasets.

## 1. Introduction

Automobiles continue to be a fundamental mean of transport worldwide. To give a figure, as reported by the AAA Foundation for Traffic Safety [1], in the US in 2019 each driver spent, on the average, about one hour per day driving covering 31.5 miles, with an increase by 5% compared to 2014. Overall, in 2019 Americans spent 70 billion hours driving, a value about 8% higher than in 2014. Considering the number of hours spent driving, the car can be seen as an ambient living. Of course, the more time drivers are at the wheel, the more potential risks they can incur.

Indeed, according to the World Health Organisation (WHO), road accidents are one of the major causes of death worldwide and the leading cause of serious injury [2]. The report shows that over 221 people die every day due to road crashes in the European Region, and thousands more are injured or disabled, with long-lasting effects.

Thus, driving safety is an important issue that is playing a very important impact in industrial and research applications, being an area that involves risks for the driver. Since some years ago, the automotive industry has started developing innovative systems called Advanced Driver Assistance Systems (ADAS) that contribute to minimise road accidents and to promote a more efficient transportation system. In general, ADAS detect various external factors such as road conditions, driver’s fatigue, the distance between cars, etc. Since human errors are among the main reasons for most road accidents [3], ADAS use sensors, cameras and other technologies to identify driver errors or nearby obstacles, and, react as necessary. Therefore, these systems are designed to improve vehicle efficiency by improving driving behaviour. It is shown that ADAS systems minimise human errors, thus reducing road accidents [4].

**Figure 1:**
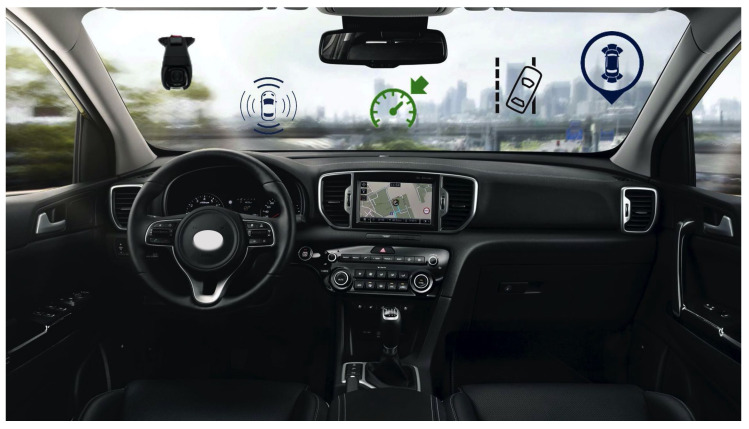
Some examples of ADAS technologies.

ADAS systems can operate passively by alerting the driver when a risk is identified or actively by reacting to avoid a possible accident [5] as shown in Figure 1.

According to the Society of Automotive Engineers (SAE), ADAS systems are classified into six levels based on the degree of automation. At level 0, only some information is provided to the driver, who has to interpret it by himself. At levels 1 and 2, the driver has to make most of the decisions by himself. In particular, at level 1 the system can control only one functionality, while at level 2 it can control more functionalities to help the driver. From level 3 to level 5, the control over the vehicle continues to increase, reaching level 5 where the vehicle is completely autonomous.

To allow the customisation of an ADAS to any driver, it is important to detect the main factors that may influence the driving style [6]. In addition, among the various factors that can influence driving style, we can consider emotions (drowsiness, fatigue, road rage, etc.) and driver conditions [7].

**Figure 2:**
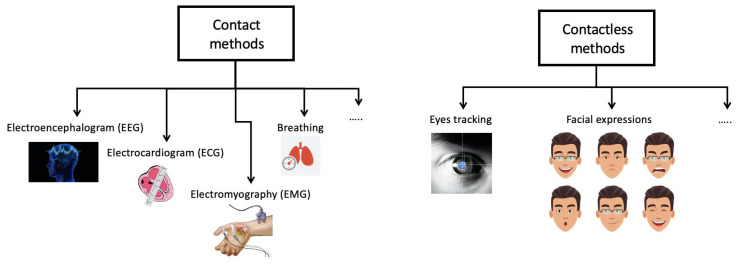
Taxonomy of methodologies for monitoring the driver’s emotions.

The development of emotion detection technologies during driving has been investigated in both academic and industrial contexts. In the industrial context, Volvo has developed Driver Alert Control [8], alerting drivers presumed to be drowsy using a dashboard camera integrated with its Lane Departure Warning System (LDWS). Mercedes-Benz has developed and introduced a Driver Alert Control system [9] that collects data from the driver’s driving style and continually verifies whether the collected information is related to steering movement and driving circumstances. Bosch has made available a driver drowsiness detection system [10] making decisions based on vehicle speed, data generated by the steering wheel sensor, use of indicators and the driver assistance camera. Lexus has developed a Driver Monitoring System for LS 600h [11]. This system makes use of a camera as well as several near-infrared LEDs to monitor the motion of the driver’s head. If it detects that the driver is not looking at the road, then the system emits a warning and briefly slows down the car. A Driver Attention Warning System has been introduced by Saab [12] utilizing two miniature infra-red cameras, one installed at the base of the driver’s A-pillar and the other in the centre of the main fascia. When the system signals the onset of drowsiness, or detects that the driver is not looking at the road ahead, the software sends a series of warnings. The driver monitoring system, developed by Subaru [13], uses an infra-red camera. If this camera detects that the driver’s eyes are not looking at the road for too long or observes signs of fatigue, a warning is emitted to restore the driver’s attention. These safety systems that detect drowsiness are not very common because they are only available in luxury vehicles. From an academic point of view some emotions such as drowsiness, fatigue, distraction, and road rage have been monitored. This monitoring is made in two ways (Figure 2):

1 Contact method: this method extracts physiological characteristics such as breathing, electrocardiogram (ECG), electroencephalogram (EEG), electromyography (EMG), etc.
2 Contactless: this method analyses head movement, facial expressions and eye tracking.

The first method measures the driver’s biological signals. The main issue is that these devices are intrusive. However, some variables can be monitored by non-intrusive sensors, e.g., those detecting seat pressure [14,15]. Using these devices, body contact is obtained thanks to the driver’s position when driving. In this case, the quality of the measurements is not as good as that achieved by using clinical instruments. This causes a performance decrease. To identify fatigue, in [16] an EEG is used to observe cardiac rhythms correlated with driver states (relaxed, alert or transitioning to drowsiness) whereas an ECG is used to examine R rhythm through a Support Vector Machine (SVM). In [17], another contact method is proposed based on breathing, observing thoracic effort. The authors detect drowsiness in drivers by analysing the variability of the respiratory signal using a chest band under real driving conditions. The main objective of the simulator tests is to collect a database of biomedical signals and drivers’ driving performance parameters in both wakeful and drowsy conditions. To classify the driver’s state, the selected signals from the simulator test are used in real driving scenarios by equipping a vehicle with other sensors such as EEG or electrooculography (EOG). The aforementioned techniques work well, with an accuracy rate of over 90% as shown in [18]. However, these methods have the disadvantage of being very intrusive, given the large number of sensors to be applied to the driver’s body.

The contactless method is based on facial feature extraction. It can use facial expression, head movement, eye closure, etc. In [19], mouth and yawning behaviours are evaluated for drowsiness measurements. A modified Viola-Jones [20] object-detection algorithm is used for face and mouth detection, avoiding the use of complex algorithms and classifiers to have a realistic implementation within an actual camera system in the car. In [21], in order to detect the pattern of long duration eye-lid closures, a standard webcam is used. When the pattern detects potential drowsiness, then it alerts the driver by a voice message. A neural network-based eye blink detector is used to locate the positions of the pupils. Recently, to solve the problem of classification, deep learning (DL) approaches, specifically Convolutional Neural Network (CNN) methods, have become more popular. In [22], an architecture using three networks is developed. The first network uses AlexNet with three fully connected layer and five CNN to detect the image feature. In the second network, to extract facial features, a 16-layered VGG-FaceNet is used. Finally, FlowImageNet is applied to obtain behaviour features. In [23], an approach for real-time drowsiness detection based on a DL method is proposed. This approach is implemented on Android applications. Here, for data processing, a Multilayer Perceptron Classifier is used.The aim is to identify the facial reference point providing the data obtained to the trained model to detect the driver’s state, achieving an accuracy of more than 80%. In [24], a contactless approach for real-time observation of driver drowsiness is presented. More specifically, several parameters related to driver physical measurements and driving behaviour in simulation are merged. To avoid large-scale disasters deriving from road accidents, a drowsiness prevention system is being developed in [25]. The authors propose a machine learning approach to improve drowsiness prediction using face recognition, eye-lid blink recognition technology, and a carbon dioxide sensor chip. In [26], the authors present a module to minimise accidents caused by driver fatigue. An algorithm is proposed to analyse both the driver’s face and eyes to measure PERCLOS (percentage of eye closure) [27], a measure of drowsiness related to slow eye closure. In [28], the driver’s smartphone is located on the windscreen of the car. Then the front camera constantly acquires the driver’s image and checks if the drivers’ eyes are closed or not using machine learning algorithms for facial expression classification. If the algorithm recognizes a possible drowsiness because the driver closes his eyes, then a timer is activated, and an alarm is generated after a certain time. This alarm is used to activate an external buzzer that is sent to the driver’s mobile phone via Bluetooth.

If drowsiness has been deeply analysed as we have previously described, this is not the case of facial expression detection with particular attention to emotions. For instance, in [29] the influence of emotions on driving is examinated. The impact of driver emotional states is studied using a vehicle simulator considering varying road conditions, inducing several emotional states: neutral, happiness, fear, or anger. The authors state that considering emotions has an impact when building a generic driving behaviour model. For instance, they argue that anger negatively influences the level of personal safety inducing a reduction of the driving performance. In [30] a non-intrusive real-time monitoring system is proposed, detecting the driver’s emotional states through facial expressions. A near infrared (NIR) camera is used to obtain the driver’s face. To obtain a set of facial landmarks, a face tracker is applied. Then, multi-class classifiers with SVM are trained with the extracted feature representations. In [31], a fast algorithm for facial expressions recognition (FER) is proposed, based a hierarchical weighted random forest (WRF) classifier capable of operating in low specification devices. First of all, facial landmarks are detected. Then, considering the spatial position between the detected landmarks, geometric features are obtained. Finally, in order to distinguish facial expressions, a hierarchical WRF classifier is proposed. In [32], a face detection model is employed to identify the driver’s face in various environmental conditions. Here, CNN are used. Two VGG16 networks are used. The first network analyses the recognized facial image and derives some appearance characteristics, while the second network identifies geometric attributes from facial landmarks. Next, these two sets of features are used together to identify emotions and, as a result, the driver is warned according to the obtained emotional state. To deal with road rage, in [33] the authors propose a real-time detection of the driver’s road rage, mainly classifying the expression of anger in real time from a live video to send an alert. To capture the frontal face, a Haar cascade file is implemented. Then, to derive facial expressions, a SVM is applied based on the K-Fold cross-validation technique. In [34], a method is proposed to capture driver aggressiveness. The proposed method considers the change of the driver’s facial emotions by exploiting an illuminator and NIR camera sensors positioned in the car. Then driver’s data are collected using a game simulator. Then, using CNN approach, a general-purpose cross-platform software library is used. First, face, left and right eye are extracted and then facial emotions are obtained. Finally, in order to classify the type of driving, a score is applied to facial emotions.

All previous approaches are input with near-frontal facial data. On the other hand, identifying road rage by using non-frontal facial expressions has not been widely studied yet. Moreover, all the systems described do not consider the influence of changing lighting conditions inside the cabin, which is a frequent event.

In this paper, an ADAS module able to automatically detect rage is proposed, alerting the driver when a predetermined level of road rage is reached. This system automatically detects drivers’ road rage level integrating a commercial and low-cost micro-camera, a face detection algorithmic step and a facial expression recognition module both based on DL algorithms that works in real time on embedded hardware platform not equipped with GPUs. Based on the classical facial expressions (“Anger”, “Disgust”, “Fear”, “Happy”, “Sad”, “Surprise”, “Neutral”), the proposed module considers two negative basic emotions, “Anger” and “Disgust”, as rage-related emotions. Finally, the decision regarding the detection of road rage is based on the temporal consistency of “Anger” and “Disgust” expressions evaluated within a sliding window. The choice to classify all facial expressions (even those not directly related to road rage) derives from the intention to provide intermediate logical blocks of the pipeline easily adaptable in the future to the detection of other driving behaviors (for example, sadness or enthusiasm, etc. ).

The remainder of this paper is structured as follows. Section 2 explains our proposed ADAS module and gives an overview of its working methodology detailing the implemented algorithmic step. The experimental setup, the considered datasets and the results are presented in Section 3. Finally, Section 4 shows both our conclusions and discussions on some ideas for future work.

## 2. Method

**Figure 3:**
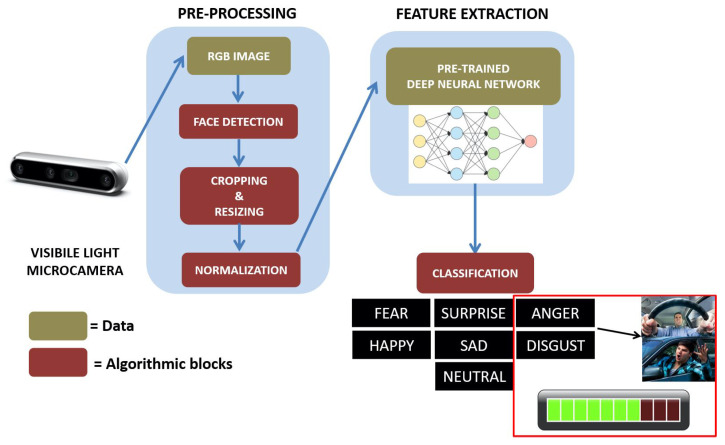
Overview of the proposed algorithmic pipeline designed and implemented for road rage detection in unconstrained scenario.

The input of the proposed ADAS module is represented by a stream of images acquired from off-the-shelf visible light micro-camera. Unlike traditional driver monitoring systems, in this work, the camera is not mounted in a specific point on the dash that always gives a full view of the driver’s face and head. Instead, it can also be positioned in points of the dash where the view of the driver’s face is not frontal, and this is an added value of the entire system as it acquires independence from the orientation of the face. The algorithmic pipeline consists of three main blocks: (1) a pre-processing stage which integrates a face detection module and a series of algorithmic steps useful to format the data for the subsequent extraction of the facial features, (2) a facial expression recognition module based on a pre-trained deep learning model, (3) a final module for the evaluation of road rage behaviour which integrates algorithms able to output an alert from the temporal analysis of facial expressions indicating the aforementioned behaviour. Each component is detailed in the following. An overview of the platform with a block diagram representation is shown in Figure 3.

### 2.1. Pre-Processing

As regards the pre-processing stage, one of the most important algorithmic step is related to the detection of a face in the streaming video acquired by the visible light micro camera, a topic still widely discussed within the scientific community of the sector. Face detection is the foundation of a road rage detection system based on evaluation of the progress of facial expressions, and the accuracy of the results has a great impact on the performance of the entire pipeline. The design scenario was considered in the proposed work, which are very far from the “ideal” conditions for acquiring the face extensively investigated in the literature. Consequently, the study of the state of the art was directed towards “Face Detection” techniques whose input consists of RGB images in which there is rarely a front view of the end-user (driver).

Face detection became mainstream in the early 2000s, when Viola and Jones [20] proposed a method to detect faces that was fast enough to run on cheap cameras. Today, it is very close to the actual standard for solving face detection tasks. In view of the limitations of the original Viola-Jones face detector in multi-pose face detection, in the considered scenario a new method that has achieved great success is proposed to solve this problem. Here the library with functions that mainly aiming real-time computer vision (i.e., last version of OpenCV) is selected. Starting from OpenCV 3.3 a deep neural network (DNN) architecture that performs an accurate face detector is included. The main advantage of this module is the ability to detect faces “in the wild” in real time even if a PC without GPU is used for the processing. Furthermore, another added value of this approach is to be able to detect the face even in less-than-ideal lighting conditions. The module is based on Single Shot MultiBox Detector (SSD) framework, using a reduced ResNet-10 model [35] and its output is constituted by the coordinates of the bounding box of the facial region accompanied by a confidence index, useful in case it is necessary to set a reliability threshold with respect to the detection of the facial region. After the detection of the face, it is necessary to crop only the facial region for subsequent analysis. In this regard, a software procedure has been implemented whose objective is to extract the coordinates of the top-left corner, the height and width of the face removing in this way all background information and image patches that are not related to the expression. Moreover, both a down-sampling step and an increasing resolution step are added depending on the resolution of the extracted facial image after cropping with the purpose to remove the variation in face size and keep the facial parts in the same pixel space. Specifically, for the down-sampling a simple linear interpolation was used, whereas a nearest-neighbor interpolation was implemented to increase the size of the facial images.

At this point as it is quite known that contrast and brightness could vary in images representing the same facial expression of a driver, it is necessary to provide a software routine within the pipeline based on a “normalization” and capable of limiting this problem to a minimum. The enhancement of the contrast in an image is generally obtained applying the equalization of histogram. However, in this way also the noise could be amplified since several pixels fall inside the same grey level range. Consequently in the proposed pipeline the normalization was obtained through the application of “contrast limited adaptive histogram equalization” (CLAHE), firstly introduced in [36]. The aforementioned methodology overcomes the limitations of global approaches by performing local contrast enhancement. The basic idea of CLAHE consists in performing the histogram equalization of non-overlapping sub-areas of the image, using interpolation to correct inconsistencies between borders. CLAHE has also two important hyperparameters: the clip limit (CL) and the number of tiles (NT). The first one (CL) is a numeric value that controls the noise amplification whereas the second (NT) is an integer value which controls the amount of non-overlapping sub-areas: based on its value, the image is divided into several (usually squared) non-overlapping regions of equal sizes. The application of CLAHE to facial region could give much better contrast and provide accurate results for FER.

### 2.2. Pre-Trained Deep Learning Architecture for FER in the Wild

Generally, in computer vision research area, there are continuous and discrete measures for emotions [37]. Complex emotions can be mapped in a two-dimensional continuous space that focuses on arousal and valence, whose values are variable between −1 and 1. On the other hand, discrete emotion theory is the claim that there is a small number of core emotions such as happy, sad, neutral, surprise, anger, disgust, and fear. Among the most important factor that determines a FER method’s performance is the use of the most discriminative features. There are two main categories of features in FER research area. Hand-crafted features are frequently used as geometric or appearance descriptor (such as Scale-Invariant Feature Transform, Local Binary Pattern, Histogram of Oriented Gradients, Gabor filter, Active Appearance Model, Active Shape Model, ⋯). The other category of feature extraction for FER is more recent and it is focused on features automatically generated by a DL architecture. From the analysis of several state-of-the-art algorithms, it is evident how CNNs have worked very well for the problem of FER in unconstrained scenario. CNN is a type of DL model inspired by the organization of animal visual cortex [38]. With CNN is possible to elaborate data that has a grid pattern, such as images, through the learning of spatial hierarchies of features (from low to high-level patterns) in automatic way.

The most considerable problem regarding the use of CNN for FER is the availability of facial expression datasets with a very high number of labelled images (asked to be in millions) with the objective to learn and extract features for obtaining high accuracies. The training of DL architectures with a limited number of images is rather challenging and sometimes it may lead to the problem of overfitting. It is also quite well known that a training process from scratch is a time-consuming task. Consequently, To address this issue one of the used solution is to evaluate the concept of “transfer learning” [39]. The recently strategy of transfer learning is based on training a specific network on a small dataset (which could be one of the main problems in the case of recognition of facial expressions of a driver) where the same is subject to a pre-trained stage on an extremely large dataset and after applied to the given task of interest. In the present work the architecture VGG16 [40] was selected for the application of transfer learning because it is widely used as the base of very recent scientific publications that have deepened the topic of FER in the wild [41,42,43].

VGG16 is a variant of the VGGNet. VGGNet is a Deep CNN that was proposed by Zisserman and Simonyan of the University of Oxford in their research work [40]. The name of this model was inspired by the name of their research group ‘Visual Geometry Group (VGG)’. It was pre-trained on ImageNet [44], which contains 1.4 million images with 1000 classes, to extract features able to distinguish one image class from another. VGG16 is structured through 13 convolution layers that are stacked together for the purpose to classify specific categories of images. For the convolution mathematical step in the aforementioned architecture a kernel of dimension 3 × 3 is used, with two different learnable parameters (*H* and *b*) that flow over the pixels *x* of each image which, results in output *y*.

The movement of the kernel is either pixelwise or skipping of several pixels and it is determined by the stride value.

A version very simplified of the convolution operation is represented by the following function:

(1) y=f(Hx+b)

The convolution layers perform an automatic feature extractor process that deduce patterns able to distinguish the facial expression class. Through the initial convolution layers only simple features are learned. These features are then combined in the later convolution layers to form more complex features. Each convolution layer is generally followed by a non-linear activation layer, Rectified Linear Unit (ReLU) to introduce uncertainty. The definition of ReLU is:

(2) ReLU(x)=max(x,0)

If the input is not less than 0, the gradient of ReLU is always 1. It has been proven that deep networks with ReLU as activation function converge faster than tanh unit and this acceleration greatly contributed to the training. Although the target task (in this case, the FER in the wild task) is different from the source (which is object classification), the low-level features that are learned by the early layers of the CNN can still be applied to most of the other vision tasks [45].

**Figure 4:**
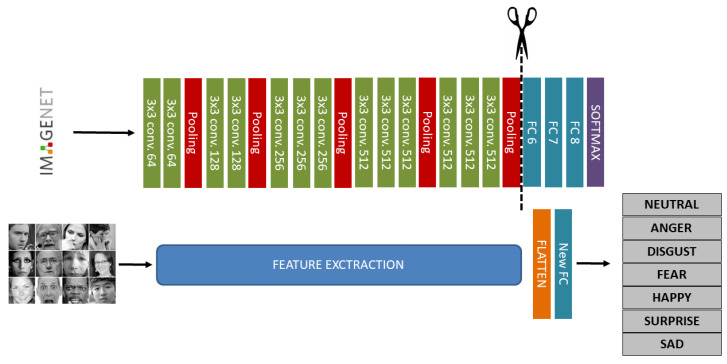
A schematic of the VGG-16 Deep Convolutional Neural Network (DCNN) architecture trained on ImageNet database. The network is 16 layers deep and can classify images into 1000 object categories (**top row**); transfer learning application for FER replacing classification layers of original VGG16 architecture (**bottom row**).

Generally, VGG16 architecture is structured into two main and well separated section: the feature extraction and the classification. In our work, considering that the feature extraction section is used for extracting new dataset features, the classification section originally structured with 3-FC layers (named FC6, FC7 and FC8) is replaced with a novel FC layer useful for tuning the desired output, i.e., the number of facial expression classes (seven classes in our case, six different facial expressions plus the neutral expression). The steps to design the proposed new architecture are shown in Figure 4:

As it is evident from the previous schematic representation, the feature extraction part is the same as the origin of VGG16 architecture but at the end a new FC layer is then added which adjusts the number of releases with the number of new dataset classes, namely 7. Between feature extraction and the new FC layer, there is a flatten layer whose function is to change the dimensions of the tensor input of the previous layer and ensure that the output size is a 1 × 1 tensor with a length corresponding to the input tensor volume.

**Figure 5:**
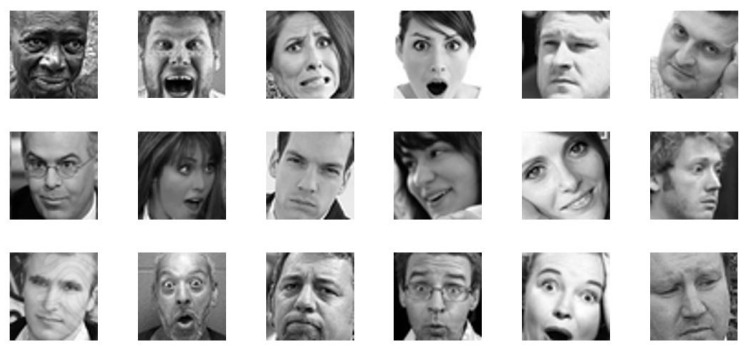
Some examples of images contained in the FER-2013 dataset.

Since it is challenging to train a complex DL architecture such as a VGG16 using only a small amount of training data without overfitting, the approach followed in the present work to tackling the aforementioned issue is inspired by recent works [46,47], which demonstrate that a fine-tuning with a small dataset performed in supervised way on a network pre-trained with a large image dataset (of different categories) can lead to significant improvement in performance. Here, the FER-2013 dataset [48] was selected for the purpose to fine-tune VGG16 architecture. FER-2013 is a large-scale dataset introduced during the ICML 2013 workshop’s facial expression recognition challenge. The dataset contains 48 × 48 pixel grey-scale images of human faces that are labelled into seven different expressions (anger, disgust, fear, happy, sad, surprise, and neutral). The dataset is structured in 3 different groups of images: (1) training set (28,709 images), (2) validation set (3589 images), and (3) test set (3589 images) (Table 1). All the images belonging to FER-2013 were extracted from the Internet. An important feature of the dataset is that the extracted faces greatly vary in age, pose and occlusion, and even the accuracy of human recognition is very low with a value around 65 ± 5% [49] (Figure 5). Actually, the CNN can now obtain accuracy on the FER-2013 task superior to human recognition. In fact, the state-of-the-art accuracy on the aforementioned dataset is 75.42%. It was obtained by combining for training stage learned feature (using CNN) and hand-crafted features [50].

In the proposed ADAS module for road rage detection, the training of VGG16 was performed with a widely used optimization algorithm, i.e., the stochastic gradient descent, that estimates the error gradient for the current state of the model using examples from the training dataset and then updates the weights of the model using the simply backpropagation of errors algorithm [51]. The backpropagation of error estimates the amount of error for which the weights of a node in the network are responsible, whereas the amount that the weights are updated during training is referred to the “learning rate” (η). In practice, the weight is scaled during training by the learning rate instead of updating with the full amount. Generally, a large value of η permits the model to find out quicker a smaller value that could enable the model to find out an additional optimum set of weights. However, this could take considerably longer time to train. A solution to find the right value of η is to consider different values of this parameter and select that one offers the best loss without sacrificing speed of training. Here, impressed by the approach described in [52], the network was trained from different values of η that are increased in exponential way, recording the training rate and training loss for each batch (fixed at 100). Only 3 values of η were considered. The experimentation demonstrate that the loss improves slowly and training of VGG16 accelerates starting with a low value of η and until the value of η becomes too large and loss goes up. In this last case the training process diverges. Moreover, it was observed that the loss function decreased fast when the learning rate was between 0.001 and 0.01. Here, we have selected η=0.001 for subsequent experiments, because through this value an excellent compromise between accuracy in classifying FER in the wild and training time is obtained.

The classifier layer of the transfer learned model (softmax classifier) is able to classify facial expressions into seven expressions classes, but an improved performance is expected when deep CNN features are provided to well established pattern classifiers. In the present study, the deep CNN features were evaluated on three different machine learning classifiers that have shown promising results in previous FER studies, such as SVM, Logistic Regression (LR) and k-nearest neighbours (kNN). SVM separates categorical data in a high dimension space finding a hyperplane with the maximum possible margin between the same hyperplane and the cases [53]. The cases that are closest to the hyperplane are called support vectors. Generally, the classes are not linearly separable and consequently a kernel function is used to perform a non-linear mapping into the feature space. Among the most widely used kernels in the literature for image classification, the Radial Basis Functions was used in the present work. LR is a predictive analysis and describes the relationship between one dependent binary variable and one/multiple independent variables. In LR, the dependent variable is binary which is contrary to linear regression having continuous dependent variable. LR can be classified as binomial, multinomial and ordinal and the model has been applied in several contexts. LR analysis is part of a category of statistical linear models which consist of fitting a LR model to an observed proportion. Here, for LR we set only the parameter *C* (that is the inverse of regularization strength λ) to 0.01 [54]. The last classifier compared is kNN that calculates in a non-parametric way the distances between the nearest k training cases and an unclassified case and classifies the latter to the majority class of the nearest k training cases. Different distance metrics can be applied in an experimental stage, with the most widely used that are the Euclidean Distance and the Manhattan distance. The two previous distances can be applied both either unweighted or with a weight. Here, k value was set to 2 and the Manhattan distance was used as a distance function [55].

### 2.3. Road Rage Detection Module

**Figure 6:**
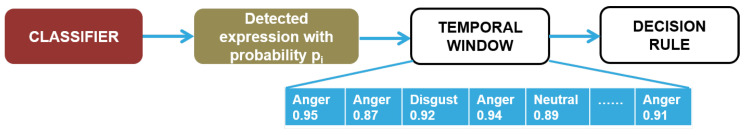
Schematic representation of road rage detection module starting from the classified facial expression labels.

The algorithmic modules described in the previous sections return a facial expression label with a sampling time of 1 second, deriving from the computational load of the algorithmic pipeline on the embedded PC used for validation. Obviously, this sampling time can vary in case of integration of the software module on a hardware platform equipped with a microcontroller. At this point it is evident that the evaluation of road rage behaviour must be done within a longer time interval than the interval between two consecutive frames acquired by the acquisition system, this to avoid false detections. Consequently, to make the whole system suitable for video sequence analysis, a decision-making strategy based on the temporal consistency of FER outcomes has been introduced (Figure 6). The decision, about the detection of road rage, is taken by analysing a temporal window of size *W* and verifying if at least *N* (N<W) frames in the window are classified as containing the facial expression “Anger” or “Disgust”. More specifically, a simple decision rule is applied in the final step of the proposed pipeline which consists of creating a buffer containing the facial expression labels, each of which is associated with a confidence index. Subsequently, a counter is incremented when one of the two considered facial expressions is detected with a confidence index higher than 0.80, and if this counter reaches the value *N* then the system returns an alert. In the actual version of road rage detection module, we set W=30 and N=20.

## 3. Results and Discussion

For the validation of the proposed ADAS module a series of experiments were conducted to verify the effectiveness of the implemented pipeline and its operation in real time. Since there are no datasets in the literature containing labelled data related to road rage behaviour and evaluated from a temporal evolution of facial expressions, for the purpose of experimental verification the performance of the proposed algorithmic pipeline were evaluated in a real context and on two literature datasets containing facial expressions acquired in the wild. Moreover, to evaluate the effectiveness of the proposed method in a context as close as possible to the real one, a dataset containing facial expressions of drivers (only with frontal view) was taken into consideration. Our experiments were performed using Python language with OpenCV 4.5.1 on an embedded PC with Intel core i5 and 8 GB of RAM. The Intel RealSense^TM^ D435 camera was used for image streaming acquisition. For DL algorithms the open-source library Keras was used, since it is a TensorFlow’s high-level API developed for implementing, training, testing, and deploying DL models [56]. The performance of FER module was evaluated using five different metrics: accuracy, sensitivity/recall, specificity, precision, and F-score. For the evaluation of these metrics, some terms are first provided in Table 2:

Accuracy (Acc) is the overall classification accuracy in term of True Positive (TP) and True Negative (TN) of the proposed method, sensitivity/recall (Se) is the TP ratio, specificity (Sp) is the TN ratio, precision (Pr) is the positive predictive rate, and F-score measures the classification performance in term of recall and precision. All the metrics are defined by the following expressions:

(3) $$\begin{array}{ccc} \mathrm{Acc} & = & \frac{\mathrm{TP}+\mathrm{TN}}{\mathrm{TP}+\mathrm{TN}+\mathrm{FP}+\mathrm{FN}} \end{array}$$

(4) $$\begin{array}{ccc} \mathrm{Se} & = & \frac{\mathrm{TP}}{\mathrm{TP}+\mathrm{FN}} \end{array}$$

(5) $$\begin{array}{ccc} \mathrm{Sp} & = & \frac{\mathrm{TN}}{\mathrm{TN}+\mathrm{FP}} \end{array}$$

(6) $$\begin{array}{ccc} \mathrm{Pr} & = & \frac{\mathrm{TP}}{\mathrm{TP}+\mathrm{FP}} \end{array}$$

(7) $$\begin{array}{ccc} F-\mathrm{score} & = & \frac{2\mathrm{TP}}{2\mathrm{TN}+\mathrm{FP}+\mathrm{FN}} \end{array}$$

### 3.1. Datasets

There are two popular “in the wild” datasets for the FER task, including RAF-DB [57] and AffectNet [58] datasets. In this study, the experiments are conducted with all of them. Moreover, the proposed algorithmic pipeline for FER were evaluated on Keimyung University Facial Expression of Drivers (KMU-FED) database containing facial expressions of different drivers captured the using a NIR camera in a real driving environment [31].

#### 3.1.1. RAF-DB

**Figure 7:**
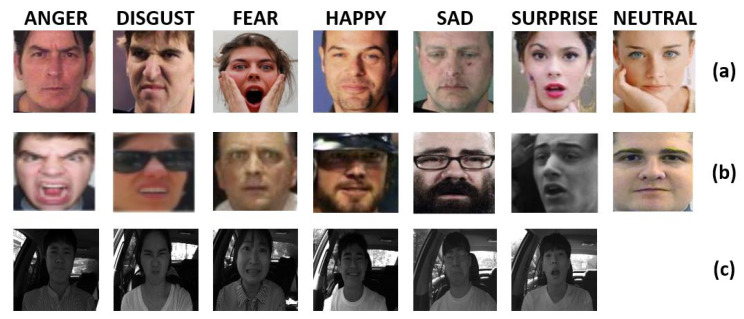
Sample images from (**a**) RAF-DB, (**b**) AffectNet and (**c**) KMU-FED datasets.

Real-world Affective Faces Database (RAF-DB) is at the date of writing of this document one of the larger existing datasets containing facial expression images. RAF-DB contains 30,000 different facial images and it is structured into a subset that contains seven classes of facial expression and in another subset that has twelve classes of expressions structured in two-tab subsets. The Images of RAF-DB are quite variable since there are expressions performed by subject of different rage, gender, and ethnicity. Moreover, the images are acquired with different head poses and illumination conditions. The database was divided into a training set (that consists of 80% images of the whole dataset) and a test set to give an objective measure of the entries. In this work only the single-label subset with seven classes of basic emotions used. This subset has 12,271 samples in the training set and 3068 in the test set. The number of samples for each emotion is given in Table 3 whereas some examples of facial images retrieved from RAF-DB dataset are shown in Figure 7a.

#### 3.1.2. AffectNet

The largest dataset for the FER in the wild is the AffectNet. It contains more than one million images that are all retrieved from the web by using keywords related to expressions. The annotation of expression for each image is done manually by trained persons. AffectNet is structured into three main subgroups of images (train set, validation set, and test set). In all the works currently present in the literature, the test set is replaced by the validation set since the test set has not yet been published. Unlike RAF-DB, AffectNet comprises eight class of facial expression (six classic facial expression plus neutral expression plus contempt expression). Here, for comparison reason with the published studies, the experiments were performed using all the images except those belonging to the “contempt” facial expression. The number of considered images divided by emotion category and by testing and training images is reported in Table 3, whereas some examples of facial images retrieved from AffectNet dataset are shown in Figure 7b.

#### 3.1.3. KMU-FED

The real-world driver facial expression images KMU-FED dataset used in this study is publicly available from the website of the Keimyung University facial expression of driver database, Korea. The dataset contains a very small number of black and white images in total (1106 images that derived from 55 image sequences of 12 different driver captured in a real vehicle driving environment). The facial images are acquired with a near-infrared camera installed on the dashboard or steering wheel. Even if the cardinality of this dataset is very low, it has been taken into consideration in the present work as it contains images very close to the considered application context. It is important to underline however that the driver’s pose is always facing the optical center of the camera. The expression labels are categorized into six classes: happy, anger, surprise, fear, sad and disgust. The dataset does not contain images with neutral expression. Some examples of facial images from the KMU-FED dataset are shown in Figure 7c, whereas the number of images considered for each category of facial expression (distinguished between training and testing images) is reported in Table 3.

### 3.2. Experiments

**Figure 8:**
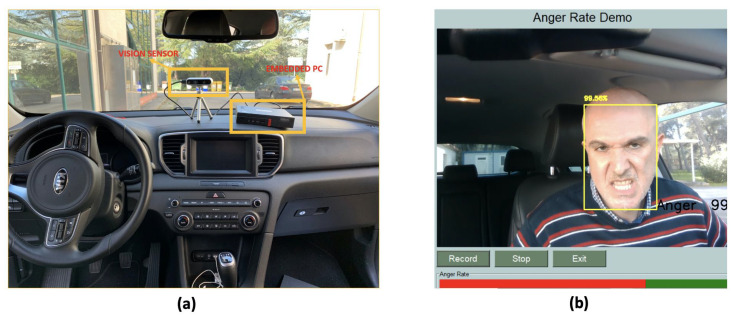
(**a**) experimental setup (**b**) a screenshot of the software application developed for road rage evaluation.

The first experiment carried out consisted in the numerical evaluation of the previously defined metrics (i.e., accuracy, sensitivity, specificity, precision, and F-score) in a real context. A sufficiently large dataset containing facial expressions of 9 drivers (6 males and 3 females) has been created. The images were captured with different light conditions typical of the passenger compartment (measured in lx-luminous emittance) both during the day with external sunlight and during the evening with external artificial light. The light intensity has been measured by an Android application (Lux light meter) running on a smartphone placed next to the human face before starting the data acquisition to estimate the passenger compartment light. Moreover, the streaming video was acquired with different head poses of the driver. The user’s head pose is captured by two angles: yaw and pitch. The angles are expressed in degrees, with values ranging from $-40^{\circ }$ to $+40^{\circ }$ for yaw angle and $-20^{\circ }$ and $+20^{\circ }$ for pitch angle. Each user involved in the experimentation was asked to simulate in sequence the six facial expressions plus the neutral expression (Figure 8a,b). The total number of collected facial images was 9 drivers × 7 expressions × 2 lighting conditions × 5 angles × 3 repetitions = 1890.

**Figure 9:**
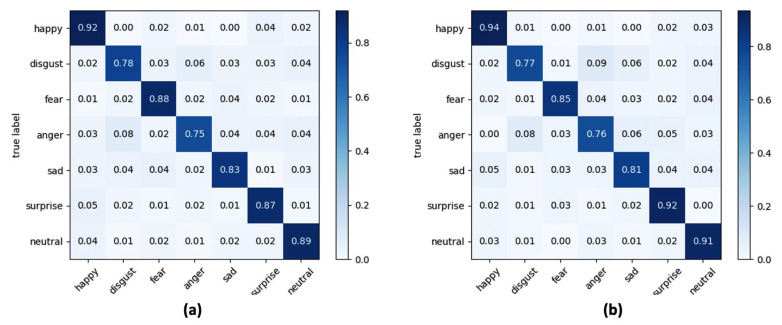
Confusion matrix for seven classes of facial expressions using SVM as classifier and at varying of (**a**) yaw angle and (**b**) pitch angle.

**Figure 10:**
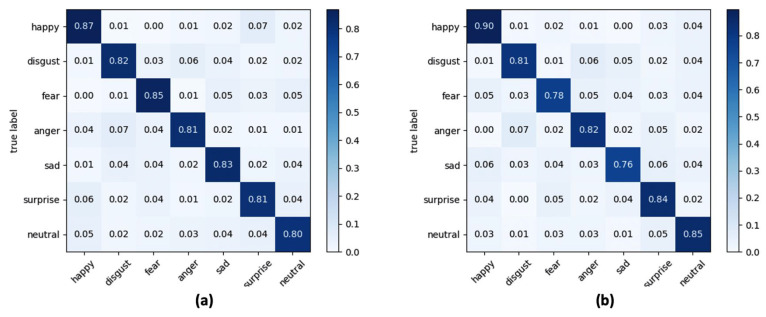
Confusion matrix for seven classes of facial expressions using LR as classifier and at varying of (**a**) yaw angle and (**b**) pitch angle.

**Figure 11:**
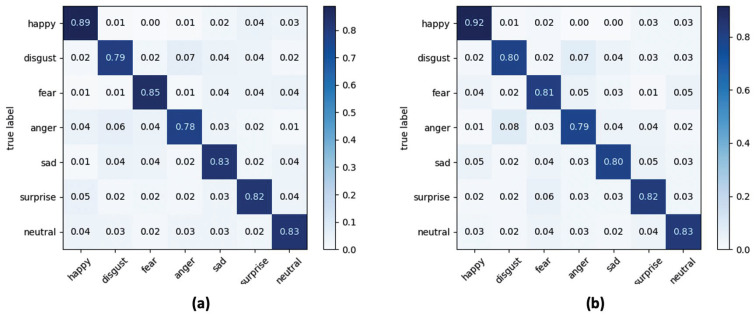
Confusion matrix for seven classes of facial expressions using kNN as classifier and at varying of (**a**) yaw angle and (**b**) pitch angle.

Since the proposed ADAS module evaluates the road rage based on the time course of two facial expressions (anger and disgust), the experiment aimed to select the best classifier that guarantees the greatest trade-off between global accuracy considering all facial expressions and accuracy in recognizing anger and disgust facial expressions. Table 4 and Table 5 report the results obtained at varying of considered classifiers (SVM, LR and kNN), lighting conditions and yaw angles (Table 4) or pitch angles (Table 5), whereas in Figure 9, Figure 10 and Figure 11 the confusion matrices of the average accuracies obtained for each considered classifier are reported.

The accuracies reported in the confusion matrices were calculated by averaging the accuracies obtained for each lighting condition and each orientation of the face considered.

The results obtained lead to a series of conclusions which are listed below. Firstly, by analysing the metrics reported in Table 4 and Table 5, the SVM classifier is the one that gives the best performance. More specifically, through SVM an improvement in the classification of about 5.8% compared to the LR classifier and of about 4.6% compared to the kNN classifier was obtained with lux = 100, while the performance in terms of accuracy with lux = 500 is less evident (+2.3% compared to LR and +2% compared to kNN). A second very important observation is related to the performance of the algorithmic pipeline with respect to the lighting conditions, which highlights how the operation of road rage module is better in the presence of low light in the passenger compartment, and this is true for every classifier tested in the experiments. From the evaluation of accuracy obtained with the variation of the orientation angle of the face it is evident how the algorithmic pipeline can obtain good classification performance in this application context. Performance is best when the driver’s face rotation occurs around the pitch angle.

In a multi-class recognition problem, as the FER one, the use of an average recognition rate (i.e., accuracy) among all the classes of considered facial expressions could be not exhaustive since there is no possibility to inspect what is the separation level, in terms of correct classifications, among classes. This aspect is of fundamental importance in the proposed road rage detection module which, as described in Section 2.3, is based on the analysis of the trend within a predefined time window of the expressions “anger” and “disgust”. Consequently, from the analysis of confusion matrices reported in Figure 9, Figure 10 and Figure 11 it emerged that the LR classifier is the one that allows a more accurate recognition of anger and disgust expressions, either by varying the pitch angle or the yaw angle. Going into a more detailed analysis on the results reported in the confusion matrices, “happy” is the expression better recognized whereas “anger” and “disgust” are the facial expressions confused the most for each classifier. At the end of the experimental session, and after a careful analysis of the trade-off between computational load, overall accuracy, and correct classification of the expressions “anger” and “disgust”, the LR classifier was integrated into the final version of the algorithmic pipeline.

### 3.3. Comparison with the State of the Art

The quality of the results can be appreciated in an ample manner by comparing the classification performance (in terms of accuracy) obtained with the proposed pipeline with other published recognition accuracy results based on a similar experimental set and performed on the datasets introduced in Section 3.1.

The accuracy reported in Table 6 and Table 7 show that the problem of recognizing facial expressions in uncontrolled contexts is still to be addressed within the scientific community. The accuracy obtained with the DL architecture proposed in this work is in line with the accuracy reported in other scientific publications for both the RAF-DB dataset and the AffectNet dataset. Regarding the RAF-DB dataset, the difference in accuracy with the work that currently reports the highest accuracy is about 2.2%, but in [59] the accuracy reported for “anger” and “disgust” is lower than the accuracy obtained through the algorithmic pipeline proposed in this paper. On the other hand, the results reported in Table 7 show that the AffectNet dataset is the one containing facial expression images acquired in largely uncontrolled contexts, but even in this case our pipeline achieves a relatively lower accuracy (about 1.8%) compared to the current state of the art. Finally, by comparing the accuracy obtained on the only dataset present in the literature and containing facial expressions of drivers, an improvement of 0.9% was obtained (Table 8). The limit of this dataset, however, is related to the fact that it contains only grey level images. Another important difference to headline is related to the computational cost of our approach with respect to the approach proposed for example in [59], in which the greater accuracies are reported. In fact, in order to integrate our system on a microcontroller, our pipeline includes deep learning architectures that work even in the absence of GPUs, and this is not the case with the architecture proposed in ref. [59] since, as shown by the authors, the experiments were performed on pc equipped with Ubuntu 18.04, with 32G RAM and using a GeForce RTX 2080 Ti GPU with 11G GPU RAM.

## 4. Conclusions

In this paper, a new road rage detection module was introduced. The innovative aspect with respect to the current state of the art is to be identified in the design, planning and implementation of an algorithmic pipeline capable of detecting the driver’s face, classifying his facial expression and evaluating the behaviour of road rage even in different contexts from the ideal one consisting of a frontal view of the face and controlled lighting conditions. To the best of our knowledge this is the first study in this research area which addresses the problem of identifying the driver’s behaviour when the orientation of the face and the lighting conditions of the passenger compartment vary. Another important aspect is that the algorithmic steps are based on DL architectures that do not require the use of GPUs and consequently the algorithmic pipeline can be easily integrated into the most popular microcontrollers in the ADAS context. It is also worth highlighting some limitations of the current pipeline. First, from the experimental setting it is evident how the lighting conditions of the passenger compartment can create problems with respect to the FER task. Furthermore, the confusion matrices show that the expressions involved in a road rage module are the most difficult to classify.

Following these critical issues, as future development the introduction within the pipeline of an algorithmic step able to standardize the facial images to be provided as input to the DL architecture proposed in this work will have to be investigated. Moreover, another improvement could be obtained using in the training stage data augmentation techniques to increase the cardinality of the facial expressions mostly used in the road rage module. A further development of the work will certainly concern the functioning test of the proposed pipeline by acquiring facial images in almost zero lighting conditions (i.e., night driving). To this end, operation will be tested both with the current used vision sensor and with any other commercial vision sensors integrating different technologies (for example specific models of IR thermal cameras). Last but not least, a further future development could be the evaluating of the performance of the entire algorithmic pipeline as the age of the driver changes, in order to search for a possible correlation between road rage behaviour and the age of the driver.

## Figures and Tables

**Table 1:** Distribution of facial expressions in the FER-2013 dataset.

Expression	Training	Validation	Testing
Anger	3995	467	491
Disgust	436	56	55
Fear	4097	496	528
Happy	7215	895	879
Sad	4830	653	594
Surprise	3171	415	416
Neutral	4965	607	626
Total	28,709	3589	3589

**Table 2:** Definition of the terms used in metrics.

Predicted Label	Actual Label	Definition
Positive	Positive	True Positive (TP)
Positive	Negative	False Positive (FP)
Negative	Positive	False Negative (FN)
Negative	Negative	True Negative (TN)

**Table 3:** Number of considered facial expression in RAF-DB, AffectNet and KMU-FED datasets, divided by training and testing.

		Facial Expression							
**Dataset**		**Anger**	**Disgust**	**Fear**	**Happy**	**Sad**	**Surprise**	**Neutral**	**Total**
RAF-DB	**Train**	705	717	281	4772	1982	1290	2524	12,271
	**Test**	162	160	74	1185	487	329	680	3068
AffectNet	**Train**	24,882	3803	6378	134,415	25,459	14,090	74,874	283,901
	**Test**	500	500	500	500	500	500	500	3500
KMU-FED	**Train**	146	70	150	160	130	150	N.A.	806
	**Test**	50	50	50	50	50	50	N.A.	300

**Table 4:** Facial expression classification results obtained at varying of three different classifiers (SVM, LR and kNN), two lighting conditions and three yaw angles.

Lx		100				500		
**Yaw Angle**		$-40^{\circ }$	$0^{\circ }$	$40^{\circ }$		$-40^{\circ }$	$0^{\circ }$	$40^{\circ }$
SVM	Acc (%)	83.86	94.27	84.57		80.60	87.13	80.34
	Se (%)	85.20	92.72	86.01		85.33	85.33	80.22
	Sp (%)	82.42	95.80	83.06		80.62	88.87	80.45
	Pr (%)	83.92	95.60	84.12		80.00	88.17	79.50
	F-score (%)	84.56	94.14	85.06		80.29	86.73	79.86
LR	Acc (%)	82.98	88.45	83.77		78.48	84.83	78.13
	Se (%)	82.04	85.40	82.81		77.04	83.11	76.25
	Sp (%)	83.96	91.74	84.77		80.04	86.68	80.22
	Pr (%)	84.22	91.79	84.88		80.60	86.96	81.14
	F-score (%)	83.11	88.48	83.83		78.78	84.99	78.62
kNN	Acc (%)	82.10	89.68	82.72		78.31	85.19	78.22
	Se (%)	83.39	87.26	83.48		77.44	83.12	77.38
	Sp (%)	80.73	92.16	81.92		79.14	87.18	79.03
	Pr (%)	82.12	91.91	82.91		78.00	86.22	78.08
	F-score (%)	82.75	89.53	83.19		77.72	84.64	77.73

**Table 5:** Facial expression classification results obtained at varying of three different classifiers (SVM, LR and kNN), two lighting conditions and three pitch angles.

Lx		100				500		
**Pitch Angle**		$-20^{\circ }$	$0^{\circ }$	$20^{\circ }$		$-20^{\circ }$	$0^{\circ }$	$20^{\circ }$
SVM	Acc (%)	86.77	94.27	85.58		80.03	87.13	79.23
	Se (%)	86.39	92.72	83.25		80.48	85.33	79.02
	Sp (%)	87.17	95.80	88.20		79.58	88.87	79.46
	Pr (%)	87.30	95.60	88.80		79.42	88.17	80.05
	F-score (%)	86.84	94.14	85.94		79.95	86.73	79.53
LR	Acc (%)	84.39	88.45	84.52		76.06	84.83	76.59
	Se (%)	84.57	85.40	82.46		75.40	83.11	74.74
	Sp (%)	84.21	91.74	86.63		76.72	86.68	78.49
	Pr (%)	84.13	91.79	86.30		76.41	86.96	78.20
	F-score (%)	84.35	88.48	84.34		75.90	84.99	76.43
kNN	Acc (%)	84.26	89.68	84.44		75.40	85.19	75.53
	Se (%)	83.96	87.26	83.07		74.67	83.12	74.20
	Sp (%)	84.55	92.16	85.87		76.13	87.18	76.84
	Pr (%)	84.18	91.91	85.98		75.87	86.22	76.02
	F-score (%)	84.07	89.53	84.50		75.27	84.64	75.10

**Table 6:** Performance comparison on RAF-DB dataset.

Methodology	Accuracy (%)
DLP-CNN [57]	74.20
FSN [60]	72.46
DDA loss [61]	79.71
ALT [62]	76.50
Separate loss [63]	77.25
PSR [59]	80.78
Our Method	**78.57**

**Table 7:** Performance comparison on AffectNet dataset.

Methodology	Accuracy (%)
PSR [59]	63.77
HERO [64]	62.11
VGG-face [65]	60.00
Facial Motion Prior Network [66]	61.52
CAKE [67]	61.70
Our Method	**61.98**

**Table 8:** Performance comparison on KMU-FED dataset.

Methodology	Accuracy (%)
LMRF [68]	93.38
Facial landmarks + WRF [31]	94.08
Our Method	**94.27**

## Data Availability

Not applicable.
